# Chimeric Antigen Receptor beyond CAR-T Cells

**DOI:** 10.3390/cancers13030404

**Published:** 2021-01-22

**Authors:** Vicky Mengfei Qin, Criselle D’Souza, Paul J. Neeson, Joe Jiang Zhu

**Affiliations:** 1 Cancer Immunology Program, Peter MacCallum Cancer Centre, Melbourne, VIC 3000, Australia; Vicky.Qin@petermac.org (V.M.Q.); Criselle.DSouza@petermac.org (C.D.); 2 Department of Clinical Pathology, University of Melbourne, Melbourne, VIC 3010, Australia; 3 Sir Peter MacCallum Department of Oncology, University of Melbourne, Melbourne, VIC 3010, Australia

**Keywords:** chimeric antigen receptor, immune cell, endodomain, combination therapy

## Abstract

**Simple Summary:**

Chimeric antigen receptors (CAR) are engineered molecules expressed on the cell surface that can recognise specific proteins and deliver an activation signal to the cells. Human T lymphocytes equipped with CAR, also called CAR-T cells, can target and kill tumour cells. This technology has been successfully used in treating some of the blood cancers in the last decade. Although the majority of research interest in CAR technology has been focused on CAR-T cells to date, the CAR design has also been used in other types of immune cells to fight against cancers. In this review, we discuss recent advances in CAR design beyond that used in conventional CAR-T cells and their novel indications to develop more potent CAR-based therapy for cancers.

**Abstract:**

Chimeric antigen receptors (CAR) are genetically engineered receptors that can recognise specific antigens and subsequently activate downstream signalling. Human T cells engineered to express a CAR, also known as CAR-T cells, can target a specific tumour antigen on the cell surface to mediate a cytotoxic response against the tumour. CAR-T cell therapy has achieved remarkable success in treating hematologic malignancies, but not in solid tumours. Currently, extensive research is being carried out to make CAR-T cells a therapy for solid tumours. To date, most of the research interest in the field has focused on cytotoxic T lymphocytes as the carrier of CAR products. However, in addition to T cells, the CAR design can be introduced in other immune cells, such as natural killer (NK)/NKT cells, γδ T cells, mucosal-associated invariant T (MAIT) cells, dendritic cells (DC), macrophages, regulatory T cells (Treg), B cells, etc. Some of the CAR-engineered immune cells, such as CAR- γδ T and CAR-NK/NK-T cells, are directly involved in the anti-tumour response, demonstrated in preclinical studies and/or clinical trials. CAR-Tregs showed promising therapeutic potential in treating autoimmune diseases. In particular, B cells engineered with chimeric receptors can be used as a platform for long-term delivery of therapeutic proteins, such as recombinant antibodies or protein replacement, in an antigen-specific manner. CAR technology is one of the most powerful engineering platforms in immunotherapy, especially for the treatment of cancers. In this review, we will discuss the recent application of the CAR design in non-CAR-T cells and future opportunities in immunotherapy.

## 1. Introduction

Adoptive cell transfer was first introduced by Steven Rosenberg in 1986 to treat cancer patients with their own immune cells [1]. In 1992, Michel Sadelain began to genetically engineer primary T cells against cancer [2]. In the following year, the first-generation chimeric antigen receptor T (CAR-T) cells were developed by Zelig Eshhar [3], although they did not persist in vivo and were not effective against cancer cells. Chimeric antigen receptors are synthetic receptors that ligate to a surface antigen and transduce the target recognition into a signalling cascade. The molecular architecture of this chimeric fusion protein comprises (1) a single-chain variable fragment (scFv) extracellular domain targeting a protein, lipid or glycan, (2) a hinge region and transmembrane domain as a membrane anchor, and (3) intracellular signalling domains [4,5].

In 2002, the second-generation of CAR-T cells was developed and proved to be effective in vitro [6] and in 2003, Dr Sadelain demonstrated that the CD19-targeted CAR-T cells could kill leukemia cells in a mouse model [7], which had an enormous impact in the future development of CAR-T therapy. Since then, CAR technology has been developed for several generations, and many innovative constructs have been introduced to improve clinical efficacy. To date, the most commonly used design in the clinic is the second-generation CAR, with either a CD28 or 4-1BB endodomain, displaying significant anti-tumour efficacy, whilst more toxicity was observed with the third generation CAR that contains both endodomains [8]. Nonetheless, this may not happen with other CAR-based cellular therapies. By converting the antigen engagement into an antibody-based binding, CARs overcome potential immune escape associated with major histocompatibility complex (MHC)-downregulation and loss of co-stimulation. This endows CAR-T cells with intrinsic anti-tumour advantages over the endogenous T cells. 

To date, CAR-T therapy has shown unprecedented success in B cell malignancies, and most patients have long-lasting complete remission [9,10]. However, antigen loss and treatment-related toxicity—cytokine release syndrome (CRS) and immune effector cell-associated neurotoxicity (ICANS)—are issues that need to be resolved [11,12]. In addition to B cell malignancies, extensive research has been done to explore the application of CAR-T therapy in solid tumours. However, to date, the anti-tumour efficacy is poor. To increase efficacy in treating solid tumours, CAR-T cells need to tackle several unique obstacles: impaired homing and trafficking, low persistence, immunosuppressive tumour microenvironment (TME) and antigen heterogeneity. To address these issues, innovative T cell engineering strategies have been developed; these have been extensively reviewed elsewhere [13,14,15]. Alternatively, promising proof-of-concept studies in conventional T cells raise the prospect of developing CAR-based approaches in non-conventional T cells, and other immune cell types to combat cancer or autoimmune diseases. In this review, we discuss recent advances in CAR design beyond that used in conventional T cells and their novel indications to develop more potent CAR-based cellular platforms in the clinic.

## 2. γδ T Cells

T cells that express heterodimeric T-cell receptors (TCRs) comprised of γ and δ chains are characterised as ‘unconventional’ T cells [16]. These cells display features of innate and adaptive immune systems. As the major circulating γδ T population, Vδ2/Vγ9 T cells recognise phosphoantigens. Indeed, aminobiphosphonates (e.g., zoledronate) are broadly used for their ex vivo expansion [17]. Other γδ T cell subsets account for rarer populations, among which Vδ1 subsets confer residency in mucosal epithelia [18]. γδ T cells are crucial players in tumour defence and distinguish stress-induced self-antigens in transformed cells. Upon ligation with TCR and/or NK cell receptors, γδ T cells target tumour cells through Th1-biased cytokines, antibody-dependent cellular cytotoxicity (ADCC), antigen presentation, and cytotoxic activity via perforin-granzyme axis [19,20]. In contrast with αβ T cells, their antigen sensing does not rely on MHC molecules; therefore, allogeneic γδ T cells could be more readily used for adoptive transfer without unwanted side effects, especially graft-versus-host disease (GvHD). 

In preclinical studies, γδ T cells were engineered with CAR to generate CAR-γδ T cells, which showed anti-tumour efficacy in leukemia models. Transduction of polyclonal γδ T cells with a CD19-targeted CAR resulted in enriched IFN-γ and TNF-α responses and systemically reduced leukemic burden in vivo [21]. A direct comparison with conventional CD19-CAR-T cells demonstrated comparable killing in aggressive leukemia xenograft but also superior cytotoxicity against leukemia cells with loss of CD19 [22]. These results illustrate that the CAR can efficiently boost anti-tumour capacity in γδ T cells, while retaining their innate cytotoxicity, providing great potential to counter antigen loss in haematological cancers. In the context of solid tumours, GD2-targeting CAR γδ T cells had equivalent lysis of neuroblastoma cells as conventional CAR-T cells, and cross-presented antigens to activate αβ T cells in vitro [23]. CAR-γδ T cells might act as professional antigen-presentation cells to induce endogenous immunity, therefore coping with antigen heterogeneity in solid tumours. However, in vivo efficacy has not yet been evaluated in neuroblastoma or other solid tumour models, and thus requires further investigation. 

CAR has also been explored in γδ T cells to minimise on-target/off-tumour toxicity. One powerful approach is combinatorial antigen sensing which controls the CAR-T cell response via a logic gate, commonly termed ‘AND-NOT’ gates. This gate provides exquisite tuning of the T cell response and is dependent on the expression of two antigens on the tumour cell surface. For example, γδ T cells recognised cognate antigen through γδ TCR and ganglioside GD-2 through DAP10-CAR, which mimics NKG2D co-stimulation. CAR-γδ T cells induced full activation only in the presence of dual antigens, resulting in equivalent killing as CD28-CD3ζ-CAR T cells against neuroblastoma and Ewings sarcoma cells [24]. This precise discrimination provides a safeguard for solid tumours that lack tumour-specific antigens. Additionally, separating co-stimulatory signals can abrogate tonic signalling, mirrored by potent effector functions and lower exhaustion marker expression [25]. This feature may prolong the in vivo persistence of the gene-modified T cells. Notwithstanding, antigen loss could compromise their cytotoxicity and consequently hinder long-term immunosurveillance. Alternatively, non-integrating transient expression of CAR in γδ T cells through mRNA electroporation has been reported to alleviate long-term toxicity while maintaining comparable potency as CAR-T cells [26]. Moreover, CAR without signalling domains can be used as a non-activating anchor to drive γδ T cell residence in proximity with malignant cells and elicit an anti-tumour response through intrinsic cytotoxicity [27]. Collectively, these examples are encouraging by leveraging temporal and spatial control on CAR-γδ T cells to develop a safe platform and retain effective anti-tumour activity.

Preclinical results have not yet demonstrated superior therapeutic efficacy, but more residual disease was observed than CAR-αβ T cells in the long-term [22]. Poor in vivo expansion and proliferation, at least in part, can be attributable to this, and are concerns that must be considered in future clinical trials. Nonetheless, CAR-γδ T cells retained TCR function and innate cytotoxicity, indicating promising effector function. It is not known what percentage of transduced γδ T cells can conserve these functional capacities after expansion in the GMP product, and this needs to be further characterised. Moreover, it also remains unclear how CAR-γδ T cells will be applied in the allogeneic setting for ‘off-the-shelf’ product. Hence, CAR-γδ T cells may not be sufficient as a stand-alone therapy. They may be used in combination with CAR-αβ T cells to alleviate immune escape. 

## 3. Regulatory T Cells

Regulatory T cells (Treg), a subset of conventional CD4+ T cells, harbour immunoregulatory properties and can be divided into two types: thymus-derived and peripheral-induced Tregs [28]. Tregs can modulate effector T cells or antigen-presenting cells through soluble anti-inflammatory mediators or cell-cell interactions [29,30]. In cancer, Treg cells behave as negative regulators in the TME, and they are central players in inducing immune tolerance to avoid tissue damage and autoimmune responses.

Synthetic biology and cell engineering has accelerated the application of CAR technology to Tregs. In preclinical studies, Tregs transduced with second-generation CARs showed antigen-specific immunosuppression of T cell responses in GvHD, solid organ transplantation, type 1 diabetes and colitis [31,32,33]. Interestingly, another study showed that CAR-Tregs elicited bystander suppression of T cells with diverse specificities [34]. This may provide widespread protection against auto-/alloreactive responses in the local environment. In other studies, CAR-Tregs were capable of inhibiting autoimmune antibody response and B cell activities [34,35]. Taken together, CAR-Tregs maintain natural immunoregulatory properties and induce peripheral tolerance. Potential cross-talk with other immune cells as indirect suppressive mechanisms might also be beneficial in highly inflammatory conditions, and thus merit further studies. 

Although CAR-Tregs convey potent immunosuppression, some studies demonstrated pro-inflammatory cytokines and antigen-specific cytotoxicity [35,36]. This may be a cause for concern about safety and stability of CAR-Tregs to generate long-term suppression. Optimising CAR-construct might be one strategy. Most studies report that the CD28 endodomain imparts superior suppressive functions in Tregs and promotes longer persistence than the 4-1BB signalling domain [37,38]. Additionally, it is not clear whether CAR-Tregs are sufficient as a single regimen to generate a durable response. In addition, their effect under established immunosuppressive therapies should be considered in different disease models. 

## 4. Mucosal Associated Invariant T Cells

Mucosal-associated invariant T cells (MAIT) are a subset of T cells that recognise vitamin B2 metabolites (5-(2-oxoethylideneamino)-6-D-ribitylaminouracil (5-OP-RU) and structural analogues) as antigens presented on non-classical molecule MR-1 [39]. These cells are highly abundant in human tissues such as the liver and can be up to 10% of CD8^+^ T cells in the peripheral blood [40]. MAIT cell responses have been studied against bacterial, fungal, and certain viral infections, as previously reviewed [41]. A few studies have demonstrated that MAIT cells play a role in cancer, either protective or pathogenic [41]. 

MAIT cells have great potential as CAR-T cells because they are MR1-restricted and also have a restricted TCR usage. MR1 is a highly conserved non-classical MHC Class 1B molecule [42]. This suggests that MAIT cells are unlikely to induce GvHD. Hence, CAR-MAIT cells could be used as universal CAR-T cells and an ‘off-the-shelf’ therapy. 

However, current strategies of isolating, expanding, and transferring MAIT cells back into patients is challenging using current CAR-T GMP production approaches. MAIT cells can be expanded ex vivo using the ligand 5-OP-RU in the presence of MR-1 expressing APCs or TLR stimulation [43,44]. As MAIT cell numbers vary in individuals (5–10% of T cells) in peripheral blood, the CAR-MAIT numbers obtained after expansion will also be limited as compared to the ex vivo expansion of all T cells. PD-1 upregulation and exhaustion phenotypes have been observed on MAIT cells in chronic viral hepatitis [45], and this may also be the case with cancers. Therefore, a combination approach with anti-PD1 may possibly overcome this hurdle. 

## 5. Tissue-Resident Memory T Cells

Tissue-resident memory T cells (TRM) are long-lived non-circulating memory T cells that persist in peripheral tissue at sites of previous antigen counter such as in infections or cancer [46]. TRM cells provide long-term immune protection through enhanced effector function against a known pathogen and recruitment of circulating T cells [46]. Due to these properties, TRM cells are an attractive option as CAR- TRM cells. CAR-TRMs could be generated against known cancer antigens. However, TRM differentiation is formed from circulating precursor cells under the influence of tissue factors, including TGF-β [47]. Therefore, the CAR-T cell product would need to have these TRM precursor cells [48]. An alternate strategy is to generate CAR-T cells with an inducible system, where once the CAR recognises the target antigen, this would in turn switch on or off genes that can convert these cells to tissue-resident CAR-T cells. Some of the genes known to drive TRM differentiation and maintenance include CD69, expression of transcription factors Blimp1, Hobit, and Runx3, and downregulation of KLF2, as reviewed elsewhere [49].

## 6. Natural Killer Cells

Natural killer cells (NK) are professional killer cells from the innate lymphoid family and have critical roles in cancer immunosurveillance. Unlike T cells, NK cells require joint signals from different activating and inhibitory receptors to target tumours [50]. They can induce rapid cytotoxicity predominantly via perforin and granzyme or through death-receptor pathways (FasL or TNF-related apoptosis-inducing ligand TRAIL-mediated) [51,52,53]. Non-MHC-restricted NK cells confer limited alloreactivity and no GvHD in adoptive transfer [54].

Despite the limited number of clinical trials, CAR-NK cells consistently show a favourable safety profile while maintaining potent reactivity. A phase I/II trial of cord blood (CB)-derived HLA-mismatched CD19-CAR-NK cells with an escalating dose was conducted in relapsed or refractory chronic lymphocytic leukemia (CLL) or non-Hodgkin’s lymphoma patients (NCT03056339) [55]. None of the patients had any side-effects, such as CRS, neurotoxicity, or GvHD, and the maximum tolerated dose was not reached. Seven out of 11 patients had complete remission, and six patients tested negative for minimal residual disease, revealing that CAR-NK cells are an effective treatment strategy against CLL. This is in line with another phase I trial for metastatic colorectal cancer with local delivery of autologous or allogeneic NKG2D-DAP12 CAR-NK cells (NCT03415100), in which all three patients significantly reduced their tumour burden with transient grade I CRS [56]. This study demonstrates the successful protection of CAR-induced killing mediated by NK cells in the context of solid tumours. Moreover, NK-92-derived CD33-CAR-NK cells were well-tolerated in a phase I trial of 3 patients with acute myeloid leukemia (AML) (NCT02944162). In contrast with CAR-T cells, repeated infusion with high-dose third-generation CAR-NK cells only caused mild elevation of CRS-related cytokines [57]. Together, these data highlight that CAR-NK cells are a safer strategy for cancer patients with a minimised risk of undesirable side effects. Additionally, different non-autologous sources were employed, including NK-92, CB-derived and allogeneic NK cells, underpinning the great therapeutic potential for NK cells coupled with CARs to provide ‘off-the-shelf’ product.

Post-infusion NK cell behaviour is vital for CAR-mediated surveillance and durable remission in patients. For example, a significantly lower early expansion of CB-derived CAR-NK cells was found in non-responding patients [55], suggesting the correlation between in vivo proliferation of CAR-NK cells with treatment responsiveness. In addition, the persistence of the CAR-NK products is still not clear, as most patients went on post-treatment due to the sign of disease progression or relapse. To prolong persistence, deleting cytokine-inducible SH2-containing protein (CIS) (a negative regulator of interleukin (IL)-15) was shown to achieve synergism with IL-15 signalling in CAR-NK cells, leading to doubled persistence and tumour eradication in lymphoma xenografts [58]. Alternatively, coupling Myd88/CD40 with ectopic expression of IL-15 in CAR-NK cells, in an attempt to mimic the Toll-like receptor (TLR) signalling, resulted in the robust proliferation and prolonged persistence in vivo [59]. As these examples illustrate, extended persistence holds promise as a means of augmenting CAR-NK cell function. However, caution is still warranted given the short-lived nature of mature NK cells and in vivo expansion, and persistence would be a major concern that impairs the anti-tumour efficacy when reaching the clinic.

Of note, CAR-NK cells exhibited efficient homing to the disease site with rapid cytotoxicity against tumours in patients. This brings about opportunities to use CAR-NK cells as a bridging therapy prior to CAR-T therapy to fulfil the demand of patients with advanced disease. With the early onset of anti-tumour activity of CAR-NK cells to lyse tumour targets, CAR-T cells may perform better with less suppression from the tumour, followed by sustained memory CAR-T repertoire to eliminate residual disease and retain immune surveillance.

## 7. Natural Killer T Cells

Natural killer T cells (NKT) are αβT cells restricted by CD1d, which is an HLA-like molecule presenting lipid antigen. NKT cells can be divided into invariant NKT cells (iNKT) recognising α-galactosylceramide (α-GalCer), and diverse NKT cells express more variant TCRs. While most studies suggest that diverse NKT cells inhibit anti-tumour immunity, iNKT cells contribute to natural tumour surveillance with immediate cytokine secretion (IFN-γ, TNF, IL-13, IL-17, IL-4, IL21, IL-22) and FasL/TRAIL pathways [60,61]. Furthermore, iNKT are clinically feasible in the allogeneic setting without GvHD; thus, universal CAR-NKT cells can be obtained from healthy donors to circumvent production and quality issues from autologous cells. Together with their effector properties, iNKT cells are an attractive platform for CAR-based therapy.

Different CAR constructs have been explored to impart iNKT cells with efficient anti-tumour properties. iNKT cells engineered with a third-generation CAR specific for ganglioside GD2 fostered expansion and persistence accompanied by superior efficacy than the second-generation counterpart in metastatic neuroblastoma xenografts without CAR-related toxicity [62]. Clinically, iNKT cells might be an optimal carrier for third-generation CAR by surmounting the unwanted side effects in CAR-T cells, and this underpins the importance of selecting co-stimulatory domains in different immune subsets. Notably, CAR-iNKT cells recapitulated the polarised T helper 1 (Th1) cytokine profile with a high IFN-γ/IL-4 ratio and increased granzyme B regardless of different endodomains [63,64]. iNKT cells naturally harbour both Th1 and Th2 cytokine profile; therefore, CAR-iNKT cells can tilt the balance towards Th1 pro-inflammatory cytokines with enhanced cytotoxic potential. These properties are favourable in attacking the tumour and could maximise their anti-tumour capacity.

To counter the lack of deep remission with conventional CARs, armoured CAR-iNKT cells have been developed and achieved marked success. Secretion of IL-15 by anti-GD2 CAR-iNKT cells improved disease-free survival with long-term persistence in bone metastasised sites and decreased the expression of exhaustion markers such as PD-1 in neuroblastoma xenograft [65]. Thus, IL-15 provides a favourable phenotype for transfer and promotes persistence, leading to greater anti-tumour potential. Based on encouraging proof-of-concept studies, a phase I trial for children with neuroblastoma is currently underway and has obtained promising safety data from initial results (NCT03294954) [66]. Alternatively, CAR iNKT cells maintained CD62L expression, acting as central memory-like cells, which enhanced their proliferative capacity and resulted in a substantial reduction in tumour burden in mice xenografted with lymphoma [67]. IL-21 in iNKT cell ex vivo expansion cultures altered the iNKT cells enhancing cytotoxicity and persistence of CD62L+ CAR-iNKT with better tumour protection [63]. Changing phenotypic composition in CAR-iNKT cells might also play a role in therapeutic efficacy as a means of prolonging persistence; nevertheless, whether this less-differentiated phenotype can be maintained over time, and help them resist T cell exhaustion, is currently unknown. Together, these data suggest superior in vivo survival, and prolonged persistence is of importance for CAR iNKT cells to harbour effective killing, but the duration of their persistence remains to be fully determined.

Building on intrinsic properties, iNKT with CARs reveals several advantages over CAR-T therapy. Rotolo et al. demonstrated that CAR-iNKT cells preserved endogenous TCR-activation in the presence of CAR-stimulation, mirrored by an improved capacity for tumour clearance in lymphoma xenografts as compared to CAR-T cells [64]. Dual targeting by CD1d and CAR as cooperative killing resulted in synergistic cytotoxicity, therefore mitigating the risk of immune escape. Different preclinical models have shown that CAR-iNKT express a higher level of chemokine receptors and exhibit augmented infiltration at the tumour lesion than CAR-T cells [62,64]. This illustrates that CAR-iNKT cells retain their physiological capacity of chemotaxis to localise to the tumour as compared to effector conventional T cells leading to better tumour control. Notably, CAR iNKT cells have shown a protective role against GvHD while their invariant TCR was intact in neuroblastoma and lymphoma xenograft [62,63,64,65]. Due to the non-polymorphic nature of CD1d, CAR-iNKT cells limit off-target reactivity, serving as a safe platform for allogeneic use. However, there are limiting aspects of iNKT cells, in that iNKT cells only account for 0.01 to 0.1% of T cells in humans and harvesting sufficient iNKT cells from an apheresis product is not feasible. Additionally, lacking a highly purified initial product would also dampen the therapeutic efficacy. Therefore, robust production of CAR-iNKT cells in the clinic still requires further investigation.

## 8. Macrophages

Macrophages as innate myeloid cells are professional phagocytes capable of orchestrating homeostasis of the adaptive immune system. Given their high abundance in many solid tumours, tumour-associated macrophages (TAM) occupy a special niche in TME, and many mediators can tailor their phenotype [68]. Immunosuppressive TAMs (M2) can dampen T cell response and facilitate tumour progression [69,70]. In contrast, M1 polarisation encompasses pro-inflammatory phenotype and harbours anti-tumour activity [71,72], thereby leading to great interest in engineering macrophages in cancer to assist immune surveillance.

CAR endows macrophages with the specificity of response against tumour-associated antigens (TAAs) in parallel with enhanced effector functions against the tumour. For example, macrophages can be engineered with CD19-CAR incorporating cytosolic domains of Megf10 or FcRγ to mimic phagocytic signalling. Consequently, this triggered antigen-specific phagocytosis and trogocytosis of lymphoma cells in an in vitro model [73]. In another study, CD3ζ-CAR macrophage also demonstrated active phagocytosis equivalent to FcRγ-CAR [74]. As such, redirected antigen-specific phagocytosis bestows spatial control and precision on eliminating cancer cells and ultimately contributes to the therapeutic effect. Furthermore, macrophages transduced with conventional CAR via adenoviral vectors polarised towards pro-inflammatory M1 phenotype and stimulated T cell responses, leading to marked tumour regression and prolonged survival in mouse models with ovarian cancer [74]. This suggests potential epitope spreading and a broader anti-tumour response propagated by CAR-macrophages within TME. Besides directly targeting tumour cells, macrophages can be transduced with CAR incorporating CD147 endodomain to express matrix metalloproteinase (MMP). This improved capacity to remodel the extracellular matrix (ECM) subsequently promoted T cell infiltration to inhibit tumour growth in breast cancer xenografts [75]. This would be beneficial for stroma-enriched solid tumours by removing physical barriers for killer cells to access tumour cells and exert cytotoxicity. CAR engineering recapitulates immune functional programs built on canonical signalling network of macrophages and therefore provides new opportunities using innate immune cells as effective CAR-carriers to treat patients with solid tumours.

As observed in the studies discussed above, CAR-macrophages have the potential to address some challenges of CAR-T cells in TME: immune cell penetration and immunosuppressive milieu. Additionally, there was evidence of cross-talk mediated by CAR-macrophages to re-educate the M2 phenotype into the M1 phenotype, facilitate maturation of dendritic cells, and cross-present antigens to activate T cells [74]. CAR-macrophages may convert the TME into an inflammatory environment and thus potentially can be used as a supportive regimen for CAR-T cells or other immunotherapies. Conversely, in vivo phenotype plasticity of macrophage should not be underestimated. There is still limited understanding as to whether CAR-macrophages can resist the suppression from regulatory cells in TME: Tregs and myeloid-derived suppressive cells (MDSC). Concerning the safety profile, there are two remaining issues. Firstly, peripheral blood-derived monocytes are highly heterogenous and manufactured CAR-macrophages could potentially develop biodistribution bias to healthy tissues with systemic administration. Secondly, macrophages have been considered as key mediators of CRS [76], thus necessitating closer attention.

## 9. Dendritic Cells

Dendritic cells (DC), a heterogeneous subset, are professional antigen-presenting cells that prime naïve T cells and reactivate memory responses. In cancer, DCs sense environmental cues in lymphoid organs or the TME and sensing of danger signals induces DC maturation leading to either immune tolerance or a tumour-specific response [77,78]. Importantly, cytotoxic CD8^+^ T cells can be activated by DCs through cross-presentation of TAAs or neoantigens to promote a stronger anti-tumour response [79]. These have key implications for cancer immunotherapy, and CAR becomes an emerging strategy to manipulate DCs for an effective response against the tumour.

Intra-tumoural DCs are considered paramount in modulating T cell functions in TME. DC engineered with CAR have been documented in a preclinical AML model to support CAR-T cells by providing immunomodulatory cytokines (activation signal 3). DC expressing a CAR containing the 4-1BB-signalling domain facilitated differentiation into the intra-tumoural DC subset, resulting in augmented cytotoxicity of infused CD33-CAR T cells with higher cytokine production and better survival in AML mice xenograft than CAR-T alone [80]. This underpins active interactions between CAR-DCs with CAR-T cells to orchestrate anti-tumour response and a synergistic therapeutic efficacy. Enhancing DC functionality may further break tolerance to tumours with the activation of bystander immune cells. On the other hand, DCs may be exploited by tumour cells or immunosuppressive mediators to subdue their function; yet evidence of CAR-DC behaviour in the TME has not been elucidated in immunocompetent models. Another topic of interest is how heterogeneous subsets of human DCs may induce different effects with CAR-T cells and their optimal ratio for patients. Notwithstanding, their clinical safety needs to be further explored, since the high level of secreted IL-12 may induce systemic toxicity [81].

## 10. B Cells

B cells and long-lived plasma cells are classically known to modulate humoral response by producing antibodies, and coordinate T cell response. They have been uncovered as active participants in tumour-draining lymph nodes, tumour-associated tertiary lymphoid structures and TME to prompt anti-tumour response, although specific subsets are polarised with pro-tumoural effects [82,83,84]. Their prevailing natures convey several advantages, making B cells attractive as a therapeutic cellular platform such as antigen-specific activation, in vivo persistence, memory pool formation and the potential to secrete proteins in large quantities.

While ex vivo manipulation of primary B cells has been limited by technical challenges, one clinical study has reported a case of CAR-transduced leukemic B cell [85]. Recently, clustered regularly interspaced short palindromic repeats (CRISPR) and CRISPR-associated protein 9 (Cas9) induced homolog-directed repair was successfully used to introduce CAR-expression cassettes into B cells. CARs equipped with the CD79β signalling domain, which is a component of B cell receptor (BCR) complex for activation, can be engineered in primary murine B cells to induce robust surface expression and antigen-recognition independent of endogenous BCR [86]. B cells could be feasible carriers for CAR-based therapy by exploiting endogenous BCR signalling, although human B cells’ functionality (e.g., proliferative capacity and antibody secretion) should be evaluated in future studies.

Considering the clinical translation, directly engineering primary human B cells and plasma cells to effectively secrete immunoglobulin and therapeutic proteins have been shown in preclinical studies to address infectious disease and protein deficiency, respectively [87,88]. In line with this concept, CAR-B cells can be used to drive the local delivery of monoclonal antibodies at the tumour site by targeting a particular TAA. This introduces the possibility of CAR-B cells as safe and controllable vehicles for releasing efficacious therapeutic antibodies that convey severe toxicity in systemic administration. Alternatively, CAR-B cells can be a novel platform for autoimmune disease and prophylactic vaccines.

## 11. The Design of CAR Constructs

The CAR construct used in conventional CAR-T cells is designed to initiate the cytotoxic killing upon antigen recognition. The application of the CAR construct in other immune subsets relies on the conserved activation pathways and/or the canonical signalling molecules in the engineered cells. Thus, the anti-tumour activities may vary in a cell type-specific manner. An optimised approach is to change the components in the CAR construct based on the activating signalling chains and inherent biological properties of engineered cells. For instance, DAP12 demonstrated superior cytotoxicity than CD3ζ in CAR-NK cells in a colorectal cancer xenograft model [56]. Distinct cellular functions beyond the cytolysis can also be induced by altering the endodomains, such as antibody production in CAR-B cells and matrix metalloproteinase secretion in CAR-macrophages. Furthermore, the extracellular domain can be derived from receptors (e.g., NKG2D) or scFv targeting non-tumour cells, such as stromal components (e.g., MDSC). The optimisation of the transmembrane domain and hinge regions may also enhance the surface expression of CAR and its functionality; however, this remains an area of ongoing investigations.

## 12. Challenges and Opportunities in Solid Tumours

To date, conventional CAR-T efficacy in solid tumours has been poor. Barriers to CAR-T cell success in solid tumours include the immunosuppressive and immune exclusive tumour microenvironment, tumour antigen heterogeneity and poor T cell trafficking. Given the distinct characteristics of the CAR immune cells described above, they may provide promising improvements in treating solid tumours in combination of conventional CAR-T cells. CAR-γδ T cells can act as professional antigen-presentation cells to cross-present antigens to conventional αβ CAR-T and endogenous T cells. These features provide great potential for CAR-γδ T cells to cope with the issue of the tumour heterogeneity. Similarly to CAR-γδ T cells, CAR-NK/NK-T cells can be used in combination with conventional CAR-T cells or as bridging therapy prior to CAR-T therapy. The superior anti-tumour efficacy and efficient trafficking capability of CAR-NK cells facilitate the early onset of anti-tumour cytotoxicity and induce the recruitment of CAR-T cells to the tumour site. CAR-DC may synergize the anti-tumour activity of the CAR-T cells, and it may also engage and activate the bystander T cells in the tumour microenvironment. Similarly, CAR-macrophage (M1) can also crosstalk with CAR-T or bystander T cells in the tumour through cytokines and chemokines, facilitating the antigen presentation and immune cell recruitment. In addition, CAR-macrophages can remodel the extracellular matrix through the production of MMP, enabling the T cell penetration into the stroma-rich solid tumours. In summary, a combination of CAR-immune cells may support the conventional CAR-T cells to overcome the current challenges in solid tumour.

## 13. Manufacture of Clinical Product

Successful ex vivo generation of CAR-immune cell products is essential for clinical application. Leukapheresis can be used for most cases. Immune subsets can be enriched by clinical grade purification through positive (e.g., NKT and γδ T cells) or negative selection (NK cells), although they represent a minority of peripheral blood lymphocytes [55,66]. These cells can be further sorted for a defined phenotype, which may have an impact on the functional activity of the final product [67]. Current expansion protocols typically incorporate engineered feeder cells and cytokine exposure (IL-2, IL-7, IL-15 and IL-21) [63,66]. Nonetheless, methodologies heavily rely on the cell type, and some expansion protocols require synthetic ligands (e.g., zoledronate, α-GalCer) [64]. Similarly, peripheral blood monocytes can be purified and induced into macrophages or dendritic cells. For example, dendritic cells in autologous cancer vaccine Sipuleucel-T can be cultured and expanded ex vivo to a high cell number (10^7^–10^9^ cells) [89,90]. In addition, induced pluripotent stem cells (iPSCs) can potentially provide an unlimited supply for CAR-immune cells, such as CAR-NK cells or CAR-macrophages [91,92]. Attractively, CAR-NK cells can also be generated from cord blood, as shown in a Phase I clinical trial [55]. Although the productivity of ex vivo generation of CAR-immune cells may be limited compared with that of conventional CAR-T cells, it is still feasible for the clinical application. As discussed in the above sections, most of the CAR-immune cells will be used in combination with conventional CAR-T cells, and the required dose will be much less than that of conventional CAR-T cells currently used in the clinic. Thus, not only the manufacturing time but also the cost of the production will be significantly reduced, which can further promote the combination immunotherapy for cancers.

## 14. Conclusions and Prospects

In the last twenty years, CAR technology has been developed for several generations; however, the conventional T lymphocytes still remain the major target for CAR engineering. Until recently, the CAR had been introduced into γδ T cells, NK/NK-T cells, DCs, macrophages and B cells, showing the great potential of CAR application in non-conventional T cells and other immune cell subsets.

The CAR technology enables host cells to specifically recognise cells expressing the target antigens on the cell surface, and subsequently trigger the downstream intracellular signalling. As the CAR design adopts the scFv from antibodies, any antigen that can be recognised by an antibody, including proteins and polysaccharides, can be used as the target for CARs. The choices of intracellular signalling are also diverse, including T-cell co-stimulatory signalling for cytotoxicity, MMP signalling for extracellular matrix remodelling, apoptosis signalling for suicide, and transcription signalling for producing specific protein, etc. (Table 1). These flexibilities facilitate distinctive approaches to target cancer cells and the tumour microenvironment by different immune cells equipped with various CARs.

**Figure 1:**
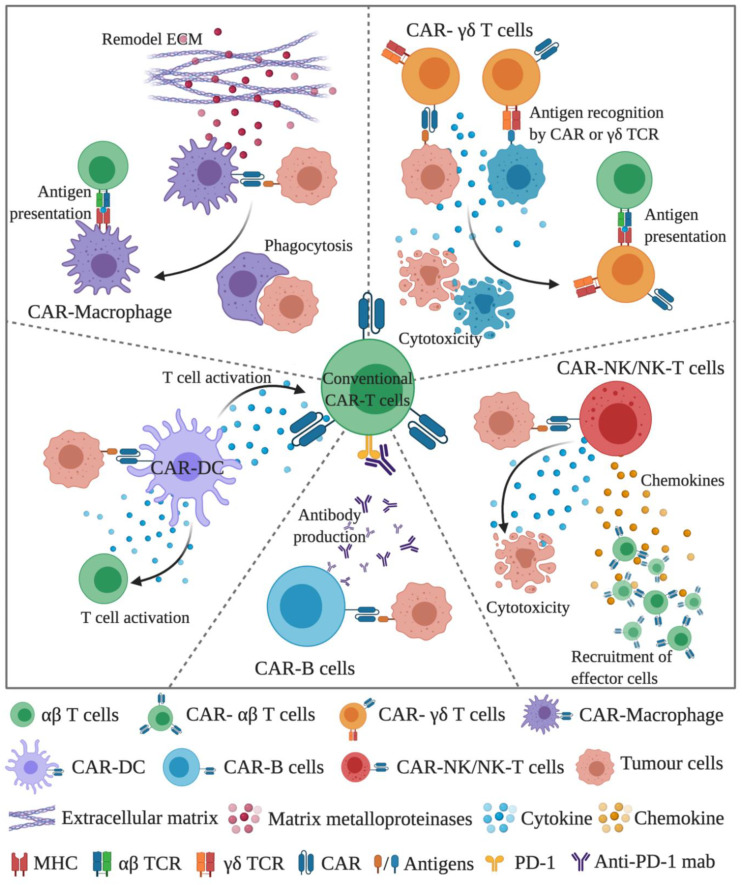
A schematic diagram demonstrating the concept of using conventional CAR-T cells in the combination of CAR-immune cells. In the combination, CAR-γδ T cells and CAR-macrophages can directly kill tumour cells and facilitate antigen presentation to cope with the tumour heterogeneity issue. CAR-macrophages can also remodel the extracellular matrix through the production of MMP. CAR-NK/NK-T cells can induce the early onset of anti-tumour activity, followed by the recruitment of CAR-T cells through chemokines. CAR-DC can support the full activation signals to CAR-T and the bystander T cells. CAR-B cells can be used as an ideal platform to deliver therapeutic antibodies, such as anti-PD1 antibody. (The figure is created with BioRender.com).

The current issues for conventional CAR-T cell therapy, especially in solid tumours, include tumour heterogeneity, trafficking, and infiltration into the tumour, in vivo activation/persistence of CAR-T cells and tumour microenvironment. Using various CAR-engineered immune cells may help to address these complex issues. One rationale for the CAR combination could be utilising conventional CAR-T cells in combination with CAR-NK cells, CAR-γδ T cells, CAR-DC, CAR-macrophages, and possibly CAR-B cells (Figure 1).

Unlike the conventional CAR-T cells, most of the intracellular signalling domains in non-conventional CAR-immune cells vary greatly depending on the host cells; for example, DAP12 for CAR-NK cells, and Megf10 for CAR-macrophages. Thus, selecting the appropriate intracellular signalling for non-conventional CAR-immune cells will be the key for the therapeutic efficacy. It should also be noted that unlike T lymphocytes, some immune cell populations are difficult or expensive to expand ex vivo, due to the low ex vivo proliferation or limited source from human peripheral blood. One possible solution is to use an alternative source to produce these CAR-engineered immune cells, for example, using induced pluripotent stem cell (iPSC) to generate CAR-NK cells or CAR-macrophages derived from iPSC or monocytes. Until now, except for CAR-NK/NK-T cells, most of the CAR-engineered immune cells are still at the preclinical stage, and some of them are even at the early proof-of-concept phase, such as CAR-MAIT and CAR-TRM cells. Further studies will be needed to explore the anti-tumour efficacy, as well as safety, of these CAR-engineered immune cells.

CAR technology is a powerful and versatile tool for immunotherapy. However, in the last few years, further modifications of the CAR itself on T cells did not achieve significant clinical improvement in solid tumours. By reviewing the pros and cons of the non-conventional CAR-immune cells, it appears promising that the combination treatment with non-conventional CAR-immune cells may overcome the major hurdles in solid cancers. Future development of the non-conventional CAR-immune cells may involve (i) customisation of appropriate intracellular signalling domain for each host cell, (ii) evaluation of the anti-tumour efficacy and safety, and (iii) optimisation of the ex vivo expansion of the non-conventional CAR-immune cells in a clinically and commercially adequate manner. Once solved, the combination CAR-immune cell therapy may become an efficient, safe, and affordable therapy for cancer treatment, and as well as other immune-related diseases.

## Figures and Tables

**Table 1:** The CAR designs are different in non-conventional T cells and other immune cell subsets.

Immune Cell Type	Extracellular scFv (Anti-)	Intracellular Domain	CAR Generation	Targeted Disease	Clinical Trials	Reference
γδ T	CD19	CD28-CD3ζ	2nd	B-ALL	-	[21,22]
	GD2	CD28-CD3ζ	2nd	Neuroblastoma	-	[23]
	MCSP	CD28-CD3ζ	2nd	Melanoma	-	[26]
	GD2	DAP10	1st	Neuroblastoma	-	[24,25]
	CD5	Non-signalling CAR	-	T-ALL	-	[27]
NKT	GD2	CD3ζ 4-1BB-CD3ζ CD28-CD3ζ CD28-4-1BB-CD3ζ	2nd and 3rd	Neuroblastoma	-	[62]
	CD19	CD28-CD3ζ CD28-OX40-CD3ζ	2nd and 3rd	B cell lymphoma	-	[64]
	CD19	4-1BB-CD3ζ	2nd	B cell lymphoma	-	[63,67]
	GD2	CD28-CD3ζ 4-1BB-CD3ζ Armoured with IL-15	2nd	Neuroblastoma	NCT03294954	[65,66]
NK	CD19	CD28-CD3ζ Armoured with IL-15	2nd	CLL, NHL	NCT03056339	[55,58]
	CD33	CD28-4-1BB-CD3ζ	3rd	AML	NCT02944162	[57]
	NKG2D	DAP12	1st	Colorectal cancer	NCT03415100	[56]
	BCMA	CD3ζ Armoured with IL15, MyD88-CD40	1st	Multiple myeloma	-	[59]
Macrophage	CD19	Megf10 FcRγ FcRγ-CD19	1st	B cell malignancies	-	[73]
	Her2	CD147	1st	Breast cancer	-	[75]
	Her2	CD3ζ	1st	Ovarian cancer	-	[74]
DC	CD33	4-1BB-CD3ζ	2nd	AML	-	[80]
B cell	Hen Egg Lysozyme	CD79β	-	-	-	[86]
Treg	CD19	4-1BB-CD3ζ	2nd	Tissue-specific immune suppression	-	[38]
	CD19	CD28-CD3ζ	2nd	Autoantibody-mediated autoimmune disease	-	[35,38]
	Factor VIII	CD28-CD3ζ	2nd	Hemophilia A	-	[34]
	HLA-A2	CD28-CD3ζ	2nd	GvHD	-	[36]

MCSP: melanoma-associated chondroitin sulfate proteoglycan; BCMA: B cell maturation antigen; B-ALL: B cell acute lymphoblastic leukemia; T-ALL: T cell acute lymphoblastic leukemia; CLL: chronic lymphocytic leukemia; NHL: non-Hodgkin’s lymphoma; AML: acute myeloid leukemia.

## Data Availability

No new data were created or analyzed in this study. Data sharing is not applicable to this article.
